# The Silent Gush: When a Jejunal Diverticulum Caused Life-Threatening Hemorrhage

**DOI:** 10.7759/cureus.89718

**Published:** 2025-08-10

**Authors:** Nikhil Batra, Santosh Hazare, Amit Kumar Meena, Amol Sathawane

**Affiliations:** 1 Department of Medical Gastroenterology, Jawaharlal Nehru Medical College, KLE Academy of Higher Education and Research (Deemed to be University), Belagavi, IND

**Keywords:** gastrointestinal bleeding, jejunal diverticulosis, nsaid enteropathy, obscure gi bleed, small bowel hemorrhage

## Abstract

Jejunal diverticula are rare anatomical abnormalities of the small intestine, often asymptomatic but capable of presenting with life-threatening gastrointestinal (GI) hemorrhage. Diagnosing obscure GI bleeding (OGIB) becomes particularly challenging when conventional modalities, such as upper and lower endoscopy or CT angiography, fail to identify the source. This is especially true in elderly patients with chronic nonsteroidal anti-inflammatory drug (NSAID) use, a known risk factor for small bowel pathology. We report the case of a 66-year-old woman with chronic NSAID use who presented with melena and hemorrhagic shock. Initial investigations, including upper endoscopy, colonoscopy, and CT angiography, were non-diagnostic. Despite aggressive resuscitation, the patient remained unstable with ongoing bleeding. Exploratory laparotomy with intraoperative enteroscopy revealed an actively bleeding jejunal diverticulum. A segmental resection of the affected small bowel was performed, followed by primary anastomosis. Postoperatively, the patient’s hemoglobin stabilized, and she was discharged after an uneventful recovery. This case underscores the importance of considering jejunal diverticulosis in the differential diagnosis of OGIB, particularly in high-risk patients. Surgical intervention with intraoperative enteroscopy can serve as both a diagnostic and life-saving therapeutic tool when less invasive approaches fail.

## Introduction

Jejunal diverticulosis is a rare and often underdiagnosed condition characterized by mucosal herniation through the mesenteric border of the small intestine. While frequently asymptomatic, complications such as diverticulitis, obstruction, perforation, and gastrointestinal (GI) bleeding can occur, with hemorrhage being among the most life-threatening presentations [1,2]. Unlike colonic diverticula, small bowel diverticula are more difficult to detect due to their deep location and intermittent bleeding pattern, often rendering standard endoscopic evaluations inconclusive [3-5].

The diagnostic challenge becomes particularly significant in elderly patients or those with risk factors such as chronic nonsteroidal anti-inflammatory drug (NSAID) use, which is associated with mucosal damage and increased intestinal permeability that may predispose to diverticular complications [6,7]. Despite advances in imaging and capsule endoscopy, obscure GI bleeding (OGIB) continues to necessitate invasive procedures such as intraoperative enteroscopy when conventional modalities fail to localize the source [8-10].

We present the case of a 66-year-old woman with hemorrhagic shock due to an actively bleeding jejunal diverticulum, managed successfully via exploratory laparotomy and segmental resection. This case highlights the diagnostic limitations of non-invasive methods, the role of NSAIDs in small bowel pathology, and the importance of early surgical intervention in unstable patients. It also emphasizes the value of a multidisciplinary approach in managing OGIB, where rapid diagnosis and treatment are critical to patient survival.

## Case presentation

A 66-year-old Indian female, homemaker by occupation, with a known history of chronic NSAID use for mechanical lower back pain secondary to degenerative lumbar spondylosis, presented to the emergency department with complaints of melena and abdominal discomfort for two days. She had no significant prior GI complaints, surgeries, or comorbid illnesses. Upon initial assessment, the patient was hemodynamically stable, with a blood pressure of 110/70 mmHg and a heart rate of 88 bpm. Her abdomen was soft and non-tender. Digital rectal examination revealed altered blood clots, suggestive of active lower gastrointestinal bleeding. 

Initial labs showed hemoglobin of 6.8 g/dL. Upper GI endoscopy (UGIE) and colonoscopy were non-revealing, except for blood in the distal bowel. Contrast-enhanced computed tomography (CECT) abdomen revealed incidental mesenteric lymphadenitis and pulmonary artery hypertension. Despite clinical suspicion of small bowel hemorrhage, digital subtraction angiography (DSA) failed to show active contrast extravasation. No coagulopathy or platelet dysfunction was detected. Differential diagnoses included angiodysplasia, NSAID-induced ulceration, and Meckel’s diverticulum. Table 1 presents the clinical course timeline.

**Table 1:** Clinical course timeline

Hospital Day	Event
Days 1–2	Melena with mild abdominal discomfort
Day 3	Presentation to the emergency department and hospital admission
Day 4	Upper gastrointestinal endoscopy (UGIE) and colonoscopy performed – inconclusive except for blood in the distal bowel
Day 5	Contrast-enhanced computed tomography (CECT) of abdomen – mesenteric lymphadenitis noted; no active source of bleeding identified
Day 6 (Morning)	Sudden hemodynamic collapse (systolic blood pressure (SBP) 70 mmHg with massive rectal bleeding
Day 6 (Later)	Emergency digital subtraction angiography (DSA) – no active bleeding detected
Day 7	Exploratory laparotomy with intraoperative enteroscopy – actively bleeding jejunal diverticulum identified and resected
Post-op Days 1–5	Uneventful postoperative period with stable hemoglobin and clinical recovery
Day 12	Discharged in stable condition with follow-up advice

**Figure 1:**
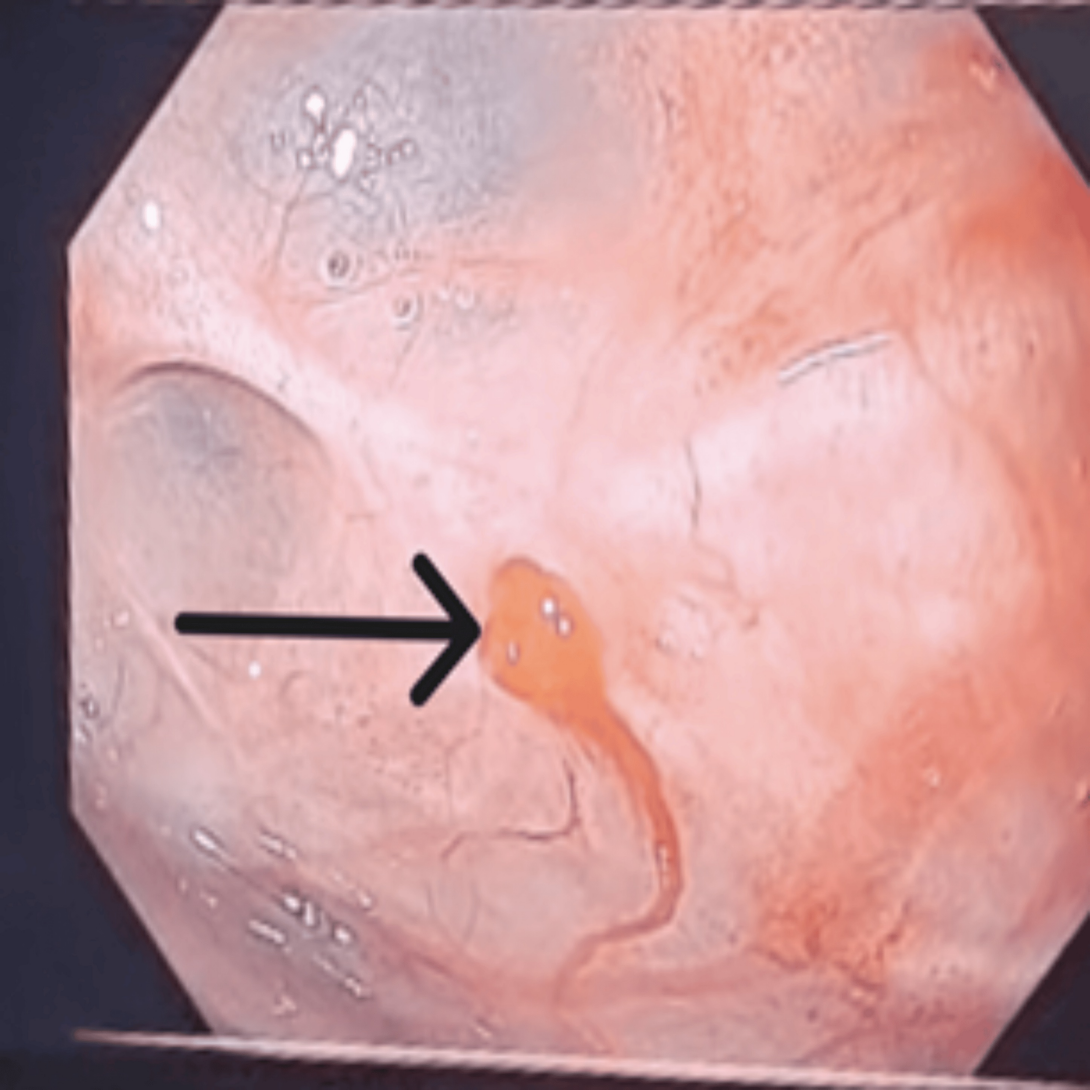
Active hemorrhage from a jejunal diverticulum, clearly visualized during exploratory laparotomy with concurrent enteroscopy Endoscopic view of the jejunal lumen showing fresh blood emanating from a diverticular orifice (arrow). Close-up visualization of the bleeding vessel within the diverticulum. The surrounding mucosa appears erythematous with signs of recent hemorrhage.

The patient was initially managed with blood transfusions and ionotropic support. On hospital day seven, the patient underwent exploratory laparotomy due to ongoing massive GI bleeding and inconclusive imaging. Intraoperative enteroscopy was performed via a small enterotomy, revealing an actively bleeding solitary jejunal diverticulum approximately 80 cm distal to the duodenojejunal flexure (Figure 1).

**Figure 2:**
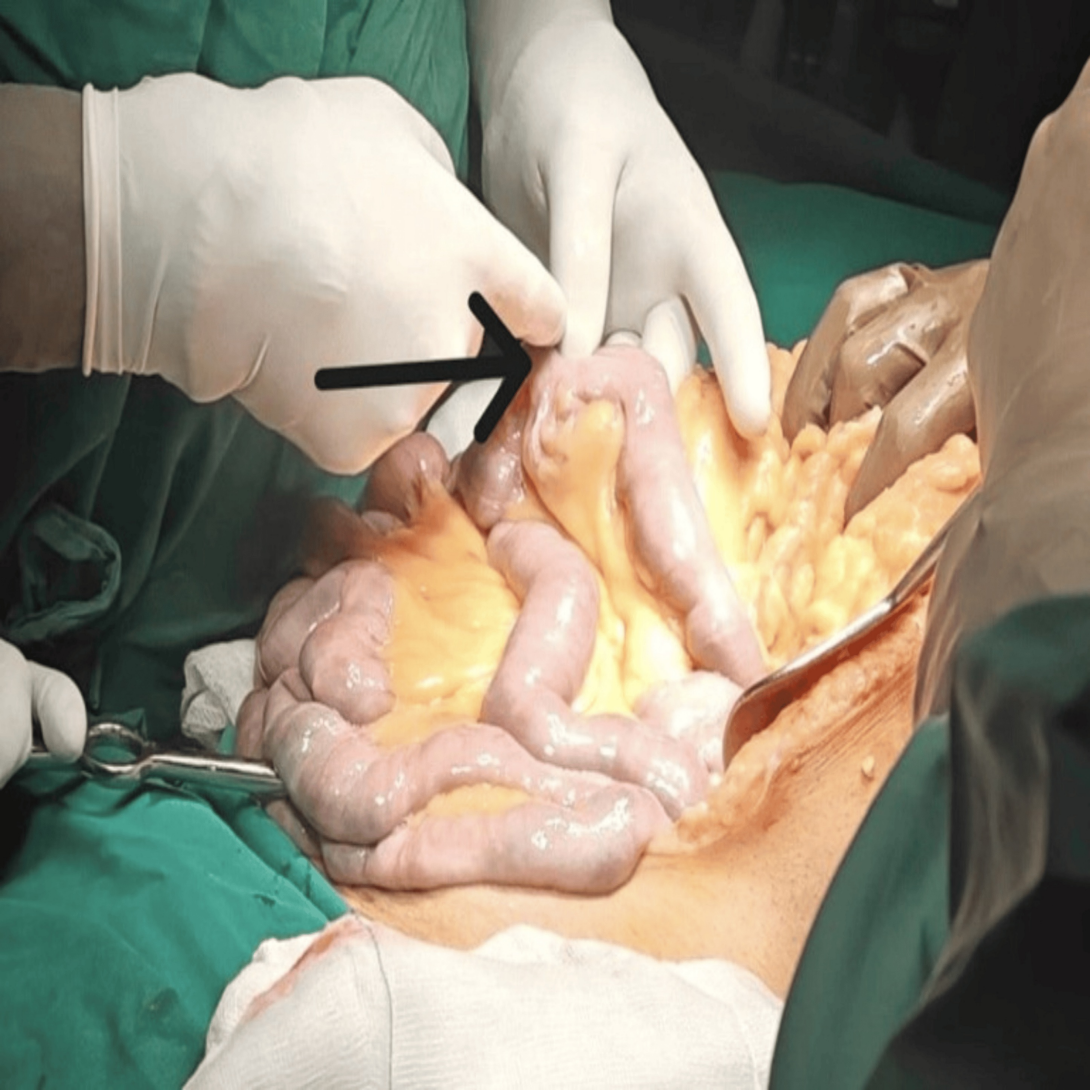
The affected jejunal segment containing the bleeding diverticulum was resected with adequate margins, followed by primary end-to-end anastomosis to restore intestinal continuity Surgical exposure of the jejunum demonstrating a diverticulum (arrow) along the mesenteric border and luminal view via intraoperative enteroscopy revealing active arterial bleeding from the diverticulum.

The affected segment was resected, and primary end-to-end anastomosis was carried out (Figure 2). Hemostasis was confirmed, and the abdomen was closed in layers. The patient tolerated the procedure well and had an uneventful postoperative recovery. Postoperative management included intravenous fluids, antibiotics, and continued transfusion support.

The patient demonstrated rapid postoperative improvement. Hemoglobin levels stabilized at 11 g/dL, and no further episodes of melena or hypotension occurred. She was discharged on day 12 in stable condition with outpatient follow-up advised. Table 2 presents a summary of the basic laboratory parameters.

**Table 2:** Summary of the basic laboratory parameters

Parameter	Preoperative	Postoperative
Hemoglobin (g/dL)	6.8	11.0
Blood urea nitrogen (BUN) (mg/dL)	32	24

## Discussion

Small intestinal diverticula are rare anatomical entities, with jejunal diverticulosis accounting for less than 1% of all GI diverticular disease [1-3]. Although often asymptomatic, complications such as hemorrhage, obstruction, diverticulitis, and perforation may occur. Among these, hemorrhage presents a significant diagnostic and therapeutic challenge, particularly in elderly patients and chronic NSAID users. This case exemplifies the difficulty in identifying a bleeding jejunal diverticulum when conventional modalities fail, reinforcing the importance of maintaining a high index of suspicion for small bowel sources in cases of OGIB with a reported annual incidence of 0.3-2.3% [4-6].

NSAID use is a well-established contributor to small bowel mucosal damage through prostaglandin inhibition and increased permeability, predisposing to ulceration or bleeding [3]. Chronic NSAID exposure in this patient likely contributed to the pathogenesis of diverticular hemorrhage. Interestingly, incidental mesenteric lymphadenitis may reflect subclinical inflammatory changes, warranting further investigation into NSAID-induced enteropathy. In addition to NSAID use, other known risk factors for jejunal diverticular bleeding include advanced age, intestinal dysmotility, chronic constipation, and connective tissue disorders, such as Ehlers-Danlos syndrome and systemic sclerosis [3].

Endoscopic evaluation is the initial approach for GI hemorrhage, yet its utility is limited in identifying small bowel lesions. Push enteroscopy extends visualization beyond the ligament of Treitz but is constrained by reach and availability [7,8]. Capsule endoscopy, though useful in stable patients, is contraindicated during active bleeding or hemodynamic instability [9]. In this patient, bidirectional endoscopy and CT angiography failed to reveal the source, highlighting diagnostic limitations reported in the literature [10,11].

Therapeutic endoscopy with injection, clipping, or electrocoagulation may be considered in stable patients with localized bleeding [12]. However, its role is limited by the difficulty in localizing jejunal lesions. Transcatheter arterial embolization is an adjunct when bleeding vessels are angiographically visible, but in our case, digital subtraction angiography was non-revealing.

This case highlights the need for a structured, stepwise approach to OGIB, integrating gastroenterology, radiology, and surgical expertise. While technological advancements improve diagnostic reach, operative intervention remains a cornerstone in unstable patients with suspected small bowel bleeding.

## Conclusions

This case underscores the importance of considering jejunal diverticular bleeding in elderly patients with unexplained GI hemorrhage, especially those with chronic NSAID use. It highlights the limitations of conventional diagnostics and affirms the value of surgical exploration and intraoperative enteroscopy as definitive diagnostic and therapeutic tools in hemodynamically unstable patients.

## References

[1] Carney BW, Khatri G, Shenoy-Bhangle AS (2019). The role of imaging in gastrointestinal bleed. Cardiovasc Diagn Ther.

[2] Gerson LB, Fidler JL, Cave DR, Leighton JA (2015). ACG clinical guideline: diagnosis and management of small bowel bleeding. Am J Gastroenterol.

[3] Bjarnason I, Takeuchi K (2009). Intestinal permeability in the pathogenesis of NSAID-induced enteropathy. J Gastroenterol.

[4] Harbi H, Kardoun N, Fendri S, Dammak N, Toumi N, Guirat A, Mzali R (2017). Jejunal diverticulitis. Review and treatment algorithm. Presse Med.

[5] Leigh N, Sullivan BJ, Anteby R, Talbert S (2020). Perforated jejunal diverticulitis: a rare but important differential in the acute abdomen. Surg Case Rep.

[6] Andersen LP, Schjoldager B, Halver B (1988). Jejunal diverticulosis in a family. Scand J Gastroenterol.

[7] Palder SB, Frey CB (1988). Jejunal diverticulosis. Arch Surg.

[8] Kim BS, Li BT, Engel A, Samra JS, Clarke S, Norton ID, Li AE (2014). Diagnosis of gastrointestinal bleeding: a practical guide for clinicians. World J Gastrointest Pathophysiol.

[9] Bond A, Smith PJ (2019). British Society of Gastroenterology: diagnosis and management of acute lower gastrointestinal bleeding. Frontline Gastroenterol.

[10] Pérez Roldán F, González Carro P, Legaz Huidobro ML (2009). Efficacy of pediatric colonoscopy used as push enteroscopy in the management of capsule endoscopy findings. Rev Esp Enferm Dig.

[11] Zhou DY, Jiang B, Yang XS (1997). Advances and applications of enteroscopy for small bowel. World J Gastroenterol.

[12] Sengupta N (2019). The role of colonoscopy and endotherapy in the management of lower gastrointestinal bleeding. Best Pract Res Clin Gastroenterol.

